# Examining the Aftereffects of Hurricane Katrina in New Orleans: A Qualitative Study of Faculty and Staff Perceptions

**DOI:** 10.1100/2012/864529

**Published:** 2012-05-02

**Authors:** Joy J. Burnham, Lisa M. Hooper

**Affiliations:** Department of Educational Studies in Psychology, Research Methodology and Counseling, The University of Alabama, P.O. Box 870231, Tuscaloosa, AL 35487-0231, USA

## Abstract

Researchers have reported how Hurricane Katrina has affected teachers who work with Kindergarten to Grade 12 (K-12), yet little is known about how the natural disaster has affected other important K-12 faculty and staff (e.g., coaches, librarians, school counselors, and cafeteria workers). Missing from the literature is the impact that this natural disaster has had on these formal (school counselors) and informal (coaches, librarians) helpers of K-12 students. Using a focus group methodology, the authors examined the aftereffects of Hurricane Katrina on 12 school employees in New Orleans, Louisiana, 18 months after the hurricane. Informed by qualitative content analysis, three emergent themes were identified: emotion-focused aftereffects, positive coping, and worry and fear. The implications for future research and promoting hope in mental health counseling are discussed.

## 1. Examining the Aftereffects of Hurricane Katrina in New Orleans: A Qualitative Study of Faculty and Staff Perceptions

Often considered “one of the most-devastating disasters in the history of the United States” [1, Paragraph 1], empirical research findings have shown the negative impact of Hurricane Katrina on school-aged children and adolescents [2–6] and adults [4–12]. Of significance, over 1 million people were relocated after Hurricane Katrina, displacing 370,000 children and adolescents in schools in Mississippi and Louisiana [10] and 200,000 children and adolescents in Louisiana alone [13]. The displacement of students from Mississippi and Louisiana resulted in transfers to schools across 46 states [4]. 

In addition to the relocated children and adolescents, 25,000 school-based Kindergarten to Grade 12 (K-12) faculty and staff in Mississippi and Louisiana were displaced [11]. Yet, despite the extensive displacement among faculty and staff in schools and the vast amount of studies about the general aftereffects of Hurricane Katrina, research that focuses on K-12 school-based employees has been limited. This paucity of research is noteworthy, considering that teachers and other school-related formal helpers (e.g., school counselors, nurses, etc.) and informal helpers (e.g., coaches, librarians, cafeteria personnel, etc.) are often among the most important care providers to school-aged children after crises [14]. Thus, it is imperative that researchers study the voices, the stories, and the lived experiences of K-12 faculty and staff after disasters such as Hurricane Katrina. The current study focuses on this understudied, important population.

## 2. Background

### 2.1. K-12  Faculty and Staff Experiences after Hurricane Katrina

A number of studies have shown the effects of Hurricane Katrina on faculty and staff in schools in Mississippi and Louisiana [3, 5, 6, 11, 13, 15], with the primary research focused on teachers rather than staff members or other faculty (e.g., librarians, school counselors). Therefore, the background for the current study draws from the empirical research examining the aftereffects of Hurricane Katrina reported by K-12 teachers. 

To understand teacher concerns after Hurricane Katrina, principals in Louisiana discussed the issues faced by teachers employed in their schools [13]. The principals reported that after Hurricane Katrina, teachers stated (1) “… higher levels of stress than in prior years” (page xvii), (2) “increased frequencies of work fatigue, job frustration, and absenteeism” (page xvii), (3) pressure with “their own personal problems resulting from the hurricanes” (page xvii), (4) a greater need for professional development because of “issues related to displacement [of students]” and more hardships “… than in the past to provide release time for teachers to attend” (page xvii), (5) more students to contend with than before Hurricane Katrina, and (6) higher numbers of absences from school. 

 The principals [13] also noted that faculty and staff had problems that were proportional to other families in the community devastated by the hurricane, (i.e., many faculty and staff also suffered from “psychological trauma” like other families did). One principal portrayed the problems faced by the teachers in the following statement:

Our staff and students were affected by the hurricane like the displaced students. My staff had to deal with personal problems that included home repair, car repair, and clearing of land on their private property. They had to work plus shuffle their time in getting their personal self and family back to a normal life [13, page 58].

The principals also conveyed administrative problems that existed in the schools in New Orleans [13]. Many of these problems could not be easily corrected such as the following: (1) “… paperwork was doubled, but no additional help was given” for the staff (page 60); (2) there was an urgent need for “… additional classroom teachers and other types of staff, such as substitutes, special education, or resource teachers, teachers' aides, support staff, and counselors or social workers” (page 65); (3) “The teachers cleaned the school themselves because the custodial staff did not return right away. They [the teachers] cleaned molded classrooms, cut grass, and mopped floors in order to get the school ready for returning students” (page 60).

Other studies confirmed that educators in New Orleans faced difficult issues in the aftermath of Hurricane Katrina [15]. There were feelings of “uncertainty” across many schools [15, page 219], as well as the “coexistence of hope and cynicism about the chances for meaningful change” in the schools [15, page 213]. Many teachers were also forced to move to new school districts when student enrollment declined after Hurricane Katrina [6]. Concerns in school districts included “personal losses and loss of significant damage to homes of employees” [6, page 343] and “lack of access to fuel and transportation routes, physical damage to buildings, equipment, supplies,…, power outages” [6, page 344].

### 2.2. K-12 Faculty and Staff Experiences after Other Hurricanes

As previously mentioned, experiences, aftereffects, and outcomes reported by K-12 faculty and staff—other than teachers—are less often documented in the literature. However, a few studies exist. While working with school counselors, Walker et al. [16] noted that after the 2008 Hurricane Ike, there was more communication with school counselors and administrators among the students and less discussion between students and teachers (i.e., more students were sent to administrators with misbehavior). Walker et al. also reported frustrations among the school counselors after the hurricane because they had fewer resources to use because of the hurricane's damage, had unmet needs for professional development (i.e., “need for educational programs that emphasize improving the student's resilience and hope in spite of obstacles” [page 156]), and needed more awareness of community “agencies that can help in our recovery” (page 156).

### 2.3. Mental Health Aftereffects after Disasters

After disasters, there are often mental health issues among the affected population. Posttraumatic stress disorder (PSTD) is frequently a focal point of discussion [12, 14], as well as anxiety, depression [14], generalized grief, and fear [4]. After Hurricane Katrina, additional mental health concerns among the victims included “grief at the loss of a loved one or home, disruptions in access to health care and medications for chronic conditions,…, [and] uncertainty regarding school for one's children” [17, page 4]. With the magnitude of Hurricane Katrina, Ursano et al. also described pervasive losses (i.e., “… losing everything that you have … losing a part of your community … and that you no longer know where to go fall all your necessities in life”), which profoundly affected people and their mental well-being [21, page 9]. Trauma-related and physical symptoms are also reported among children and adults after disasters [6].

### 2.4. Aftereffects of Disasters: Grief, Traumatic Grief, and Resiliency

Faculty, staff, and other diverse populations have long reported the scope and magnitude of grief up to and including traumatic grief following a range of disasters (e.g., natural, human-made, etc.). For example, O'Mallon [18] stated, “Global events such as earthquakes, tsunamis, the 9/11 attack, and Hurricane Katrina fall into the category of traumatic grief. Grief associated with loss of life, home, and finances left families vulnerable …” (paragraph 12). Although the authors of the current study agree that disasters can engender traumatic grief, we do not believe that human beings are fated to experience traumatic grief following disasters. Nonetheless, the empirical research suggests that the possibility exits. Thus, it is important to understand the variability and types of grief reported and how the grief process may relate to coping after disasters. Toward this end, Kübler-Ross' [19] five stages of grief may provide a framework to make meaning of the aftereffects and outcomes often reported by individuals who experience hurricanes, disasters, and other traumatic and adverse events. 

Kübler-Ross' [19] introduced five stages of grief (i.e., denial, anger, bargaining, depression, and acceptance) following death. Overtime, the five stages of grief have also been used to explain the range of reactions and possibly the “phases” that people move through following various losses (e.g., natural disasters, death, loss of income, and divorce, to name a few). 

*Stage 1 (Denial).* Denial leads to initial comments such as “No, not me, it cannot be true” [19, page 33]. Denial is usually a “temporary defense” and transition into “partial acceptance” [19, page 34]. During denial, the initial shock caused by the loss is experienced. *Stage 2 (Anger).* According to Kübler-Ross, denial “is replaced by feelings of anger, rage, envy, and resentment” (page 43). At this stage, the grieving person questions “Why me?” (page 43). He or she is “… very difficult to cope with from the point of view of family and staff … anger is displaced in all directions and projected onto the environment at times almost at random” (page 43). *Stage 3 (Bargaining).* Kübler-Ross illustrated the grieving person's mentality during this stage as “If we have been unable to face the sad facts in the first period and have been angry at people and God in the second phase, maybe we can succeed in entering into some sort of an agreement which may postpone the inevitable happening” (page 71). Kübler-Ross also described a sense of guilt among the grieving during this stage. *Stage 4 (Depression).* According to Kübler-Ross [19], when the illness or issue cannot be ignored, “… numbness or stoicism … anger and rage will soon be replaced with a sense of great loss” (page 75). Kübler-Ross noted two types of depression, reactive depression and preparatory depression. During reactive depression, the grieving individual “… has much to share and requires many verbal interactions and often active interventions” (page 77). In contrast, during preparatory depression, “there is no or little need for words. It is much more a feeling that can be mutually expressed and is often done better with a touch of a hand, a stroking of the hair, or just a silent sitting together. That is,… when he [or she] begins to occupy himself [herself] with things ahead rather than behind” (page 77). *Stage 5 (Acceptance).* With “enough time (i.e., not a sudden, unexpected death),” and “… help in working through the previously described stages, he [she] will reach a stage during which he [she] is neither depressed nor angry about his [her] fate” (page 101). This stage is “almost void of feelings” (page 102).

### 2.5. Resiliency

Resiliency among children and adults affected by disasters is often discussed in the literature [10, 17] and has been acknowledged by the helping professions. For example, after Hurricane Katrina, Ursano et al. [17] urged psychiatrists to “keep in mind that many people we see are going to be resilient” (page 10). Because of the power of resilience after disasters, the American Psychological Association [20] has suggested that psychologists build and support resilience skills among their clients (i.e., suggesting that clients interact with both “family and friends” who were and were not involved in the disaster, develop the view that “change is … an ongoing experience,” and retain “a hopeful outlook”) (paragraph 6). 

Resiliency literature has a positive stance. For instance, Ursano et al. [17] put forward the view that “loss and exposure to trauma are not necessarily risk factors in themselves” (page 10). To add, mental health practitioners have been challenged by Gheytanchi et al. [7] to reconsider older mental health interventions and to take into account interventions that “focus on functional recovery rather than psychopathology” (page 126), such as psychological first aid [21]. PFA has a resilience focus and aims to be culturally sensitive to all ages and developmental levels.

## 3. The Present Study

In-depth knowledge about the experiences of an understudied diverse group such as K-12 faculty after Hurricane Katrina can lead to better means to support, intervene, and counsel school employees after natural disasters. To further this knowledge, this study examined the mental health aftereffects, coping strategies, and outcomes (i.e., relational, environmental, financial) of K-12 faculty and staff who served students before Hurricane Katrina and then returned to the schools after Hurricane Katrina. While one study assessed Mississippi faculty and staff after Hurricane Katrina [6], the present study focused exclusively on faculty and staff from New Orleans, Louisiana. The perceptions of K-12 school employees (faculty and staff) were investigated 18 months after Hurricane Katrina. Because we sought to uncover an in-depth, rich knowledge of the participants' perceptions, we used a focus group methodology. 

Four overall research questions informed our study. They were as follows (1) What, if any, *symptoms*, *concerns*, or *effects* have been experienced since Hurricane Katrina? (2) What, if any, coping* strategies* were used, if any, to manage the symptoms, concerns, or effects of Hurricane Katrina? (3) In what ways, if any, have significant *relationships* (relationship with family, school personnel, students, God) evidenced before Hurricane Katrina changed after Hurricane Katrina? (4) To what extent, if any, has the *environment* (e.g., working conditions, neighborhood) changed after Hurricane Katrina?

## 4. Method

### 4.1. Participants

Faculty and staff recruited for this study were employed in nine private schools in the greater New Orleans area in Louisiana. Faculty of all grade levels and subjects (i.e., athletic coaches, librarians, school counselors, learning specialists, and K-12 teachers) and staff (i.e., secretaries, kitchen staff, maintenance staff, and resource managers) were invited to participate. Interested potential study participants responded to an E-mail invitation, so that they could be contacted. Of the people contacted, 12 people (eight staff and four faculty) from three schools agreed to be a part of the study. The final study sample consisted of eight females and four males. Racial background included 11 who identified as White American and one who identified as African American. The mean age of the participants was 29.8. The study took place in February 2007, 18 months after the Hurricane Katrina devastation of New Orleans, Louisiana.

### 4.2. Instrumentation

#### 4.2.1. Demographic Data Sheet

The participants were asked to complete a demographic data sheet before the start of the focus groups. The sheet required participants to indicate their age, gender, racial background, and occupation, as well as how long they have lived in the Greater New Orleans area, whether they are living in the same home that they lived in pre-Hurricane Katrina, and, if not, where they are currently living.

### 4.3. Focus Group Interview Guide

We developed a focus group interview guide that listed six topics and related questions. *Coping.* Let us start by talking about things you have done to take care of yourself (to cope) since Hurricane Katrina. *Working Conditions (Environmental).* Everyone has an important job that they do in the school. We are interested in better understanding how your job has changed since Hurricane Katrina? *Aftereffects (Signs and Symptoms).* First consider how you were feeling initially (i.e., right after Hurricane Katrina)? Then consider how you were feeling 1 year later—let us say on the anniversary of Hurricane Katrina? How are you feeling now? *Proximal/Distal/Environmental*: When the hurricane hit, where were you? Throughout the evacuation, what were your major concerns? Where did you go? *Taking Care of Self (Coping).* How was your ability to take care of yourself before Hurricane Katrina? What is your ability to take care of yourself now following Hurricane Katrina? *Health Care Providers Help (Coping)*: Has anyone sought out emotional or mental health professional help since Hurricane Katrina?

### 4.4. Procedure

After we obtained Institutional Review Board (IRB) approval for the study and the focus groups guide, we sought written approval to conduct the study from the headmaster of a K-12 school in New Orleans, where the focus groups would be held. On the day of the data collection, we and a graduate assistant were present at the school.

Four focus groups were completed. We began by introducing ourselves to the participants and provided an introduction to the study. Specifically, we explained the ground rules for the session, assured the participants that what was to be talked about in the group was confidential, and discussed the audio recording methods to be used. We gave each participant an informed consent form and a demographic data sheet. Once consent was received from all participants, the digital recorders were turned on to record the sessions, and the focus groups began. Participants were provided a notepad and a pen to write down thoughts or comments during the focus groups.

Time limits were kept to ensure that each topic and question was explored. Ample time was given to allow each participant to voice his or her opinion, experience, and story. At the end of the focus groups, participants were thanked for their time and participation and were given a copy of the informed consent signed by the researchers, as well as a list of resources (i.e., public mental health agencies, crisis hotline) in the greater New Orleans area.

## 5. Data Analysis

Audio recordings were transcribed verbatim by two graduate-level research assistants. Procedures for qualitative content analysis were used [22–24]. Initial qualitative codes were considered from the empirical and theoretical literature examining the aftereffects of disasters among K-12 teachers. We conducted several reviews of all transcripts and uncovered recurrent and unique codes and concepts evinced in the data (i.e., text). We used an iterative process to refine the codes and to create a study codebook. Consistent with content analysis, we performed a line-by-line analysis of the study transcripts. Several additional readings were completed to ensure that no codes were left out from previous readings. When consensus of the coding structure [25] was achieved among all research team members, responses were reviewed for preliminary themes and patterns. The code words were then divided into nine preliminary themes, or *salient *themes [22–24]. Patterns and salient themes evidenced from this process were then considered across the four focus groups. The analyses led to the emergence of nine preliminary themes or subcategories. Some of the themes were combined. After these coding analyses were completed, three major themes emerged among members in all groups. The three themes were then compared to Kübler-Ross' [19] stages of grief.

## 6. Results

From the data analyses, nine preliminary themes emerged: emotions, depression, positive/improvement, worry/fear, weak/tired, shock, angry, survival, and change. After all analyses were completed, three major themes emerged: Emotion-Focused Outcomes and Aftereffects, Coping and Positive Steps Toward Improvement, and Worry and Fear. 

The three themes—and subthemes—that emerged suggested that K-12 faculty and staff suffered greatly from Hurricane Katrina. The negative aftereffects were evident—even 18 months after the disaster—in Theme 1. Theme 2 illustrated the resiliency and coping efforts on the part of the participants, despite the setbacks and extensive, far-reaching losses after Hurricane Katrina. Theme 3 illustrated present fears and worries and questions about the future. The following sections present the major themes, subthemes, and comments.

**Theme 1 (Emotion-Focused Outcomes/Aftereffects)**

Theme 1 can be best explained by four distinct subthemes for the faculty and staff including Shock and Denial, Anger, Guilt, and Depression. From our analysis, we believe that the faculty and staff precisely described three of Kübler-Ross' [19, 26] stages of grief (i.e., denial, anger, and depression) in Theme 1. In addition, the faculty and staff also talked openly about guilt, which falls under Kübler-Ross' bargaining stage. Thus, four of the five stages described by Kübler-Ross were evident in this first theme.

**Subtheme 1.1 [Shock and Denial]**

The faculty and staff described initial shock and denial after the onset of Hurricane Katrina's damage. Consonant with Kübler-Ross' framework, the participants experienced shock caused by the losses, and comments were wrought with initial intense feelings of disbelief. Also, fitting with Kübler-Ross' [19] framework, the denial experienced by the participants appeared to be a “temporary defense” or transition into “partial acceptance” [26, page 34]. Comments related to shock and denial were heard

- “Who would have thought that within 24 hours your entire school, your church, your temple, your community is gone?”
- “It just seemed like a bad dream. It was unreal.”
- “I mean the first week after was just shock, with an underlying despair.”
- “Initially, disbelieving, confused, sense of loss … bewildered.”
- “I think originally I was in denial. My husband and I were both in denial when we saw the news.”

**Subtheme 1.2 [Anger]**

The Anger subtheme reflected the participants' frustration toward people and agencies. This subtheme paralleled Kübler-Ross' [19] second stage of grief, anger, whereby the denial stage “… is replaced by feelings of anger, rage, envy, and resentment” (page 43). Just as Kübler-Ross posited, “… anger is displaced in all directions and projected onto the environment at times almost at random” (page 43). The anger after Hurricane Katrina, expressed in the focus groups among the educators, was toward numerous entities (e.g., school administration, politicians, the federal government, and media). Several teachers also talked about their distressing feelings about receiving relief from the Red Cross Relief. See illustrations of anger

- “You know, teachers who had been here the longest like 30 years were let go, and everybody was mourning. I felt this tension inside that made it worse … and, it was a slap and shock, and we're still mourning our missing colleagues.”
- “I cannot read a newspaper today. Just, whatever's in it just fills me with rage, with the political systems, and the missing money, and everything.”
- “The rest of the world has moved on, but we're still here.”
- “I don't think that we can rely on the federal government to fix our problems.”
- “I want people to know that it's not over … for those of us who lost a lot, it's not over.”

**Subtheme 1.3 [Guilt]**

Although Kübler-Ross' [19, 26] did not have a stage called guilt, she acknowledged the significance of guilt in the bargaining stage. In this study, the bargaining stage [19, 26] was not as straightforward. From the focus groups, the guilt category involved the schemas and guilt feelings the K-12 faculty and staff reported after Hurricane Katrina. “Survivor's guilt” appeared to be prevalent among the focus group members who had little damage from the hurricane and/or subsequent flooding. Among the educators, several described guilt

- “I do remember feeling guilt … because we were in places where we were fine … And then, to, to see … on TV what was happening. And, and I think roughly a thousand people they're saying … died …. I do remember feeling guilt.”
- “We were lucky too, we lost our, a lot of our roof and our chimney so we had the gaping hole, we didn't have the flood. And we felt the survivor, non-flooder guilt….”

**Subtheme 1.4 [Depression]**

The Depression subtheme involved sadness, despair, helplessness, and hopelessness as illustrated in the following statements. This subtheme was also quite similar to Kübler-Ross' [19, 26] fourth stage of grief, depression. Just as Kübler-Ross noted, when the issue (i.e., Hurricane Katrina's damage) could not be ignored, “… anger and rage [was] … replaced with a sense of great loss” (page 75). The following illustrate depression

- “I received financial assistance. I was eligible for food stamps and all kinds of things and the food stamps were so hard for me to accept. I cried the first day that I used them. I just stood at the register and cried.”
- “I'm, acutely aware of the need to grieve, and that it's a long process; it doesn't happen overnight, and no one can tell anyone how long one should be in that process.”
- “… numb was the overwhelming feeling that I heard quite a bit, disappointed and a little depressed and sad.”
- “I mean there are people that are just stuck in this helpless state, and they don't know what to do."
- “… I lost 30 years worth of (teaching) material that I collected.”
- “… I don't know what I have and don't have. I've replaced a whole lot and people have donated stuff. It was very difficult for me personally because I had lost huge portions of my life both professionally and personally and that's been really, really hard.”

**Theme 2 (Coping and Positive Steps Toward Improvement)**

The second major theme was Coping and Positive Steps Toward Improvement. This theme involved five subthemes for the faculty and staff: Coping, School, Gaining Control, Connected Community, and Change.

**Subtheme 2.1 [Coping]**

The subtheme of Coping does not fit clearly into any of Kübler-Ross' grief stages. It involves both positive and negative means to cope in the aftermath of Hurricane Katrina. Among the educators, coping mechanisms varied. There was positive coping among discussions (i.e., physical exercise and rebuilding the schools and homes). This category also involved introspection. At least three participants mentioned their own and others' “self-medicating” to cope with the hurricane effects. A few teachers discussed other unhealthy coping habits after the disaster, including smoking, overeating, oversleeping, and using alcohol

- “Self-medicating, pretty good down here you know. I'll never forget to when I was in Houston and I called some of my family that lived on the North Shore. And I said look, I'm in, I'm coming back, I'm just a week later, I'm leaving Houston. What do you need? Generators, shovels, axes, chainsaws, what do you need? The response was a case of vodka, case of gin, two cases of wine …”
- “I drank more … I mean I was never drunk … I would go home and have a drink every single night and you kind of needed it to chill out and that lasted quite a while and if you talked to people … all kinds of people were doing the same thing.”
- “I used an old prescription of Vicodin.”

**Subtheme 2.2 [School]**

The school subtheme relates to the needs of the faculty and staff, as well as those of students. Since this subtheme is twofold (faculty/staff and students), we first considered how rebuilding the school met the needs of the faculty and staff, followed by how rebuilding the school met the needs of the students.

**Faculty and Staff Needs Met with School**

Based on numerous conversations, getting the schools opened and in order after Hurricane Katrina was a positive goal for many of the faculty and staff in this study and a step toward overall improvement in the quality of life. In fact, many educators set out to get the school back in order as soon as they could return safely. The following comment illustrates the priority and importance of school in the lives of the teachers: “I was in the school as soon as I could get my boots through without touching the [flood] water … I felt like I was doing something.”

While some classroom teachers longed to get back with the children in the classroom to “feel back to normal,” other faculty and staff described a preference for “physical labor.” At least three males in the study talked about laying sod, which seemed to represent improvement, stability, and starting over. The striking differences in how the educators contributed to the startup of the school in the aftermath (i.e., certain teachers expressed longing to work with the children, while others preferred involvement in labor or “completing tasks” [e.g., laying sod, painting, cleaning, and discarding]) was revealing of their own personal needs. Many agreed, either implicitly and explicitly, that returning to school was a way to gain a sense of normalcy

- “For me the normalcy of being in the classroom, being with the kids was great because the rest of my life was such a mess …. I lost a lot and it was just nice… the normalcy and the stability because nothing else was predictable.”
- “One of the things that I've always found is that when there is crisis in my life that one of the best things for me to do is to be here … be at school and with kids. I had 8.5 feet of water in my house, but it was nice to be here and be with the kids and colleagues.”
- “I wanted nothing to do with the kids. I wanted to do physical labor … to me that was kind of putting back the school.”
- “We did things like cleaning up the school … some people laid sod.”
- “I wanted to do physical labor, to me that was kind of putting back the school. I wanted to paint and sod. I just felt that needed to be done … there was really nobody on campus to do that sort of thing. So I came in two 12-day stretches in October and November.”
- “It was just the physical … trying to put back the school in some way, shape, or form.”

**Student Needs Met with School**

Based on the responses in the focus groups, we also saw that many faculty and staff realized that the school represented a haven for the children. Based on the focus groups, the educators aimed to get the school back to normal as soon as possible to meet the needs of the students.

Once the schools reopened, the teachers ensured that the curriculum and hurricane-related activities and discussions were completed. This aspect of the study correlated with Prinstein et al. [14] views on what is required for students to cope appropriately after disasters. According to Prinstein et al., three types of coping assistance are needed by children in the wake of disasters: emotional processing, reinstitution of familiar roles and routines, and distraction. Based on the focus groups, we believe that all three types of coping assistance were provided. The teachers offered the first type of coping assistance (emotional processing) through drawing, writing, and the curriculum “In the fall after Katrina … the curriculum was organized around the storm.”

The second type of coping assistance (reinstitution of familiar roles and routines [14]) was the most vivid of the three types in teacher conversations. Teacher responses included the desire to get the students back to the schools to have “normalcy.” Comments included the following.

- “I think the school, for a lack of better of a word, is the same now as far as interactions and how the school runs. Things are kind of back to normal.”
- “… well, definitely working with the kids is very, very normal. I don't think the kids sense any change.”
- “The kids were changed. I think they were just so grateful to be back to have this stability. For the most part, they did not see the chaos behind the lines. To them it was the school, except for a few changes, it was the school they had.”

The third type of coping (distraction) was in place. According to Prinstein et al., distraction is aided by supportive relationships including peer, parental, and educators and the ability to “process a traumatic event through relevant discussions, reenactment activities … or playing games related to the disaster” (page 465). This was evident in the following statement. “Everyone wrote memoirs, we read stuff that was storm-related. When the kids came back in January, they did not want to talk about it.”

**Subtheme 2.3 [Gaining Control]**

The Gaining Control subtheme relates to how the faculty and staff coped and had a sense of improvement. The means of gaining control involved such activities as exercising, helping others, journaling, going to counseling, returning to the school, and seeking spiritual guidance. We found that the faculty and staff learned ways to help themselves and others. This appeared to be an integral step toward accepting their reality after Hurricane Katrina

- “The first thing I did was exercise …. I joined a gym and you know, I felt a sense of normalcy … because I usually exercise.”
- “Like I think that probably digging down deep and coming up with the fact that you've just got to move on. You learn, you just learn to do that; become stronger.”
- “Perseverance and helping others, and opening our home to others, help you get through the initial shock. Returning to a routine was a big thing.”
- “… The first method of coping has really been spiritual; seeking guidance and trusting in God.”

**Subtheme 2.4 [Connected Community]**

Many faculty and staff discussed the desire to feel connected with the community after the hurricane. This was illustrated by various participants

- “Another thing I did was I got involved … only by E-mail but in a lot of the citizen's groups and I was emailing people and writing letters and doing that kind of thing. I did a lot of that and that felt good.”
- “And one of the things you did that was really nice is that you gathered people in. You did that and that helped me a lot because you were constantly gathering people in …. I tried to help other people.”
- “I guess in the beginning it was sort of like perseverance, that you just have to go on, and keep on pushing to get things done.”
- “So, just like perseverance and helping others and opening our home, home to others, helps you get through the initial shock.”
- “I said, pray, plant sod, pretty things up, remove water lines, think positively, and to help others.”

**Subtheme 2.5 [Change]**

The Change subtheme was one of the most distinctive and appeared to be closest to Kübler-Ross' final stage of grief, acceptance. Kübler-Ross [19] described this stage as “… neither depressed nor angry about his [her] “fate”” (page 101). The faculty and staff spoke often about the “new normal,” reflecting a level of acceptance and openness about their destiny. The statements were somber, realistic, and profound

- “We felt more fragile a year after the storm than we did … and in conversations we try to put our finger on it, it is because things are getting back to a new normal in New Orleans, and we have to figure out what that new normal is.”
- “All of us are still struggling with, do we stick it out. In many ways the city has returned to some semblance of normalcy, although it is a new normal, it's nothing that will ever be normal again.”
- “… just returning to a normal routine was a, a big thing. Uh, and then, you know, just focusing on the positive …”
- “In many ways the city has returned to some semblance of normalcy, although it's a new normal, it's, nothing will ever be normal again. It's a new kind of normal.”
- “I figure 5 years away to feeling anything like normal. It's not anywhere near over for me, not even close to being over.”

Laying the sod and seeing the green growth of the grass was also symbolic, especially for the men in the focus groups. The green sod appeared to represent a fresh start and getting the school back to the “new normal.” Comments, such as the following, offered hopefulness and reframing, as the faculty and staff focused on “seeing green” instead of the damage from the storm

- “Little things we take for granted. You know that weeds grow in the sidewalk, and you don't want them … you welcome that weed. Salt water came in here and killed a lot of trees and everything.”
- “When I got back, I would drive to a different part of the city every day. And this was an oasis coming here. Because we started planting sod, this was the only green part for miles around.”
- “Things had become somewhat normal after school started. It was green out here. It was greener than it had ever been. We had planted sod where we didn't have grass grow before. This was an oasis, because you would feel depressed between here and home.”

The change category also highlighted a level of optimism among the faculty and staff. The following comments highlighted positive feelings, acceptance, and growth despite the vast setbacks the faculty and staff had faced over the past 18 months

- “I think that my prestorm mental health is just starting to come back to where it was prestorm.”
- “I think now…. 18 months post-K I feel like our family is beginning to return to normal …”
- “Somewhat optimistic. Um, there's a chance.”

**Theme 3 (Worry and Fear Now and in the Future)**

The third major theme involves two subthemes: Uncertainty and Overwhelmed. This final produced present feelings, thoughts, and concerns about the future.

**Subtheme 3.1 [Uncertainty]**

The Uncertainty subtheme reveals worries of the faculty and staff. The following comments demonstrate that the faculty and staff worried about the unknown not only at home, but also at school

- “I worry about the future … still.”
- “Nobody knows what the future brings.”
- “I don't know what the future holds.”
- “I was worried about people that I knew and I was worried about my house.”
- “… I don't even know what I have now. Before I could tell you what I had …. now that uncertainty is there and I'm probably hyper sensitive to it because I got the double whammy of home and school. I was putting my classroom together and taking my house apart … it was challenging.”

The faculty and staff also indicated that the dynamics with the students and the parents had changed. Teachers described how increased levels of attention were needed by the students and their parents after Hurricane Katrina. This was reflected in the following comments

- “I think that has put more pressure on us in the work force.”
- “Yeah, with the little ones … we have little ones who get totally freaked out if there is a storm.”
- “Not so much our little ones because they don't remember enough but some of the older lower school kids see a storm coming and they panic because they think it's a hurricane.”
- “... I think I get many more phone calls about, what are teachers expecting, um, I think I get many more phone calls about how their kids are feeling, whether they're self conscious or not. Um, more phone calls about homework, and how much homework they're doing, or why didn't they make this grade on a test. And so, they're, they're much more, not making their kids as resilient maybe as they should be, and learn how to deal with … adversity. And I find that much more stressful.”

**Subtheme 3.2 [Overwhelmed]**

The Overwhelmed subtheme was evident among the faculty and staff. For example, as the educators contemplated their future, some conversations pertained to whether or not they will stay in New Orleans for the long term.

- “We're all wondering, really, is this where we want to be?”
- “… all of us are still struggling with, do we stick it out?”

The educators also expressed feelings of numbness.

- “I am still very numb in a lot of ways. I don't have real highs and I don't have real lows. … there's just so many don't knows.”
- “Well my, my first word would be, you said numb, stunned…”
- “… during that time it was really uh, disbelief uh, sadness. But, but I think more, more of the numbness.”

The fears and frustrations since the storm were still prevalent

- “I just put down three emotions. Frightened, angry, complacent yet hopeful.”
- “You know, its fear of so many things if you … if you, really let yourself feel it.”
- “I'm terrified of the crime.”
- “I was so glad to get the anniversary over with because the media buildup was so overwhelming.”
- “My life will be defined as the first 41 years as pre-K and hopefully the next 45 years post-K. It defines every conversation. I run into people I haven't seen in two years for whatever reason … how did you do in the storm first question they ask. It still defines our lives every day …”

## 7. Discussion

There were important findings from this study after “one of the most-devastating disasters” [1, paragraph 1] of our time. Beyond the emergence of three major themes (Emotion-focused Aftereffects, Coping, and Worry and Fear), this study offered insight about what faculty and staff from New Orleans experienced, how they coped, and documented increased stress at home and school. This study emphasized that while faculty and staff are often unselfish frontline responders to get their schools back in order after disasters, they also suffer their own losses, setbacks, and need healthy coping skills and interventions. While many faculty and staff in this study suffered pervasive losses [17], this study also revealed optimism and hope about rebuilding.

This study revealed a number of consistent findings in the literature. In particular, much of the results related to Kübler-Ross' [19] stages of grief. We posit that the educators went through three to four of the stages described by Kübler-Ross (i.e., denial, anger, bargaining, and depression). Consistent with the findings of Ward and Shelley [6], many faculty and staff also suffered greatly because of “personal losses and loss of/significant damage” to their own houses and belongings (page 343). 

As revealed by the principals in Pane et al. [13] and confirmed by the teachers in this study, stress levels rose after the hurricane. The teachers reported “increased frequencies of work fatigue, job frustration, and absenteeism” as they dealt with “their own personal problems resulting from the hurricanes” [13, page xvii]. Similar to Pane et al., many of the faculty and staff in this study also volunteered to assist with such tasks laying sod, cleaning floors, painting, removing mold, and discarding ruined items to get the school repaired and ready for the return of students. There was a sense of urgency among the faculty and staff to get the school back in shape. 

The faculty and staff can be commended for efforts taken after Hurricane Katrina. First, after disasters, children need to return to school as soon as feasible to reestablish a routine [3, 6]. There was substantial evidence among faculty and staff that reopening the schools was a priority in New Orleans. Second, the teachers should be commended for helping the children cope after the schools reopened and for providing a sense of normalcy at school. The teachers appeared to implement the coping assistance suggestions of Prinstein et al. [14]. 

The faculty and staff talked openly about the need to reconnect with family, friends, neighbors, and the schools after Hurricane Katrina. This pattern follows Weems et al. [12] who posited after examining post-Hurricane Katrina victims in Mississippi and Louisiana that “positive social support” will “likely foster a quicker resolution of psychological problems” (page 2304). These were also other similarities to the literature (i.e., signs of “resiliency” among the educators [10, 17, 20], “uncertainty,” [15, page 219], and “coexistence of hope and cynicism” [15, page 218]).

This study revealed a number of contrasts to the literature. For example, the unhealthy means of coping (e.g., self-medicating, numerous discussions of alcohol to cope) have not been discussed in other studies with faculty and staff, to our knowledge. Also, the “change” subtheme revealed a discussion of the “new normal” in New Orleans. This discussion was unique as the faculty and staff moved toward acceptance of their plight. In addition, the heightened optimism that was shown, with the participants “seeing green” and talking about the “new normal” in New Orleans instead of the damage from the storm, seemed to be distinctive. Overall, faculty and staff were realistic about their concerns, frustrations, and fears about the future and some had lingering questions about whether or not to stay in New Orleans.

There were limitations to this study. First, this study had a small number of participants in the greater New Orleans area. Second, the study included participants from three private schools and no public schools, which makes generalizability unknown. Third, there were design limitations (i.e., we did not address triangulations and did not use in-depth interviews).

There are implications for policy and/or clinical practice. This study underlines that school administrators and mental health practitioners need to be “… sensitive to the problems experienced by teachers” [13, page 70] in postdisaster situations. This particular study shed light on the importance of extending Pane et al. from “teachers” to *all* faculty and staff. Clinical practitioners should review the comparisons with Kübler-Ross [19] and consider how these stages can assist them and their clients in post-disaster situations.

Further examination of Prinstein et al. [14] coping assistance model and its use with children following disasters is merited. Whether the use of such coping assistance lessens depression or posttraumatic stress disorder symptoms should be explored in future studies. Additionally, as Prinstein et al. suggested, more investigation of the “frequency and utility of various types of coping assistance at different points in the recovery process” (page 473) would be helpful for understanding how teachers and other school-related staff can effectively meet children's emotional and psychological needs after future disasters. 

Current school policies related to disasters and crises should be reviewed by all school systems. Additionally, crisis intervention plans should be in place and reviewed frequently. Because disasters impact everyone affiliated with schools (i.e., administrators, faculty and staff, parents, stakeholders, communities, children, and adolescents), postdisaster plans and support mechanisms should be developed for all educators, staff, and students. While initial support may include clothes, food, shelter, and medical attention, preparing for mental health concerns and providing access to mental health services to help people cope effectively are vital in post-disaster conditions.
